# Ecologies of care: qualitative insights from a psychosocial study on veterinarians’ experiences in Northeastern Italy

**DOI:** 10.3389/fvets.2025.1650809

**Published:** 2025-12-04

**Authors:** Ciro De Vincenzo, Ines Testoni

**Affiliations:** 1 Department FISPPA, University of Padova, Padova, Italy; 2 Departmento SPGI, University of Padova, Padova, Italy

**Keywords:** veterinarians, mental health, qualitative research, ecological approach, psychosocial stress, reflexive thematic analysis, professional identity, Italy

## Abstract

Over the last decade, literature has raised concerns about the psychological well-being of veterinarians, highlighting exposure to economic, organizational, and clinical stressors. However, most research is quantitative and concentrated in Anglophone contexts (UK/US), leaving a significant gap in qualitative, EU-based data. While quantitative research highlights trends, qualitative methods offer in-depth insight crucial for developing tailored interventions. Addressing this gap, this study adopts a qualitative methodology informed by a psychosocial framework to explore the lived experiences of 20 companion animal veterinarians in Northeastern Italy. Semi-structured interviews were conducted and analyzed using Reflexive Thematic Analysis. Five themes were generated, revealing that veterinary science and veterinarians are undergoing a profound transformation within their evolving sociocultural role and mandate. Findings highlight tensions related to fragmented communities, socioemotional delegitimization, and the hidden weight of changing client dynamics. This ripple effect, marked by complex tensions affecting personal (expectations) and professional (caring function) domains, challenges the coherence and sustainability of their occupational identity and psychosocial well-being, requiring an ongoing labor of readaptation. The study concludes that contemporary psychosocial distress in veterinary work should not be understood solely through individual-level factors. Rather, distress reflects profound changes in the sociocultural landscape, including shifts in how human–animal relationships are framed and changing client expectations. This highlights a pressing need for veterinary science to engage in deeper dialog with the human and social sciences. An ecological perspective is essential for designing targeted systemic interventions, informing policy, and creating effective, context-sensitive training.

## 1 Introduction

Over the past decade, a growing body of international research has highlighted alarming levels of psychological distress among veterinarians (1–4). While most findings derive from U. S. and U. K. regions or from other anglophone countries (5), trends of the phenomenon seem to show global patterns. While some factors are different, also veterinary students are experiencing similar and particular stressful experiences to their well-being (6–10). Mental health disorders have become increasingly prevalent, with veterinarians exhibiting some of the highest reported levels of burnout, anxiety, depression, and suicide risk among healthcare professionals (11–16). Empirical evidence confirms that veterinarians face a significantly elevated suicide risk compared to the general population (17–20), with recent studies linking this to moral injury, secondary traumatic stress, and emotional fatigue (21–25). This elevated risk has prompted extensive research into the specific factors that contribute to psychological distress within the veterinary profession, revealing a complex interplay of occupational stressors, personality factors, and environmental conditions. A complex and multi-layered pattern of factors contributes to these mental health challenges (26, 27). Among the most frequently cited stressors are high workloads, the emotional demands of client interactions, exposure to animal suffering, unresolved grief, moral dilemmas, communication and the routine administration of euthanasia (28–33). Veterinarians are increasingly required to navigate not only clinical tasks, but also the psychological terrain of clients’ expectations, grief, and decision-making—dimensions often unaddressed in their formal training (34–37). End-of-life care, in particular, has emerged as one of the most emotionally taxing aspects of veterinary practice (38). Veterinarians frequently act as mediators between animal suffering and client distress, making difficult ethical decisions while containing both their own and their clients’ emotional responses (39–41). Euthanasia-related moral distress, affective overexposure, and the gap between professional ideals and institutional constraints contribute to what some describe as a chronic erosion of emotional well-being (42). Veterinarians often find themselves working not only with animal patients, but with emotionally burdened owners, as well as under constant scrutiny in a setting that is more consumer oriented. A study involving over 9,000 U. S. veterinarians found that high levels of neuroticism and poor work-life balance were significant predictors of burnout, depression, and suicidal ideation (43). An Italian study showed an association between poor reflective functioning and substance abuse in relation to suicidal ideation (44). Conversely, a supportive work environment and meaningful professional identity were identified as protective factors (45). Although some research has focused on personality traits such as neuroticism, perfectionism, or insecure attachment as predictors of psychological distress (46), the field is increasingly shifting toward a systemic and contextual understanding of veterinary mental health. Scholars are now paying greater attention to work environment, organizational culture, client relationships, gendered expectations, and moral uncertainty (47, 48). Importantly, gender differences are emerging as a recurring theme, with women reporting significantly higher levels of emotional exhaustion, compassion fatigue, and reluctance to seek help (49, 50). Early-career professionals and veterinary students also represent high-risk groups, often entering the profession with strong vocational ideals but facing emotional disillusionment when confronted with the realities of clinical work (51, 52). Recent international studies have stressed the need for cross-disciplinary approaches and the development of tools that integrate psychological, veterinary, organizational, and critical lenses. This includes adapting concepts such as moral distress, burnout, vicarious trauma, and disenfranchised grief—well-studied in human healthcare—to the veterinary context (53–56). Yet, in countries like Italy, this line of inquiry remains underdeveloped.

Despite the increasing recognition of mental health challenges within the veterinary field, existing literature relies heavily on quantitative surveys. While these studies provide valuable statistical insights, they fail to fully capture the lived experiences of veterinarians. Due to this, research in Italy remains limited, leaving significant gap in understanding the unique experiences of Italian veterinarians. This study aims to address this gap by employing a qualitative research to explore veterinarians’ experiences in Italy. Through reflexive thematic analysis of semi-structured interviews with *N* = 20 veterinarians, this research will provide a nuanced understanding of veterinarians’ perspectives on personal, relational, organizational, and sociocultural challenges in their contemporary practice. The findings will contribute to the current literature on veterinary well-being and mental health, offering insights to inform tailored supporting interventions and targeted policy changes at several levels (57). Thus, this research intended to address a significant gap in the Italian context, where existing studies are scarce (10, 28, 35, 36, 44, 49, 50), and qualitative explorations building on veterinarians’ perspective are even more limited. Additionally, the study expected to contribute to international literature with thick, psychological informed analysis that responds to calls for a more ecological and translational approach to the field, that is strategic knowledge that take into account contextual dimensions and inspire practical implications (58).

## 2 Materials and methods

The study aimed to foreground the voices of Italian veterinarians working with companion animals, offering nuanced psychological insights and understanding of how they experience and make sense of the emotional, relational, and structural challenges embedded in their daily practice. The empirical research question guiding this inquiry was: *how do medical veterinarians represent, make sense of, and live their contemporary clinical practice?*

### 2.1 Methodology and research design

In light of the aim and research questions, the present study adopted a qualitative methodological framework (59, 60) to research in veterinary medicine (58). Although a heterogeneous field of diversified approaches (61), qualitative methodology supports the exploration or the deep examination of new, undervalued insights, offering thick, rich descriptions and bringing to existing literature exploratory lines of research or additional comparative data (62). Thus, qualitative methods allow an “emic,” participant-centered, closed, and intimate approach to empirical data, supporting a nuanced, first-person understanding of participants’ experiences (63).

This study was further grounded into social and cultural psychological theories, specifically the sociocultural psychological theory (58), meaning that participants are considered as active, unique social and semiotic agents (64) constantly engaged in meaning-making processes to structure and make sense of their reality (65). Moreover, social psychology endorses an ecological understanding of human experiences, promoting researchers’ capability to contextualize individuals’ experiences within and across different social spheres. Coherently with social and cultural theorization in contemporary psychology, thus, the individual is approached as ontologically being interconnected, intertwined, intersected with their contexts and environment and the nature of their unique experiences is always linked to practical, material interactions and relationships. This theoretical perspective implies that the subjective experience does not grow out of nowhere but rather takes a (unique) shape across the ongoing interactions with bigger social and cultural environments (the clinic, the family, the community of professionals, the regulations and so on).

The manuscript follows the COREQ (COnsolidated criteria for REporting Qualitative research) guidelines for qualitative studies (66).

### 2.2 Data collection method

Consistent with the aim, research question, and theoretical–epistemological background mentioned above, the study adopted semi-structured interviews as the key strategy for data collection (67). In particular, semi-structured interviews are appropriate and suited for qualitative research, employing a combination of pre-defined questions, functioning as a guide and derived from existing studies or researcher’s previous knowledge, and the emergence of novel, unpredictable issues from participants’ narratives (68). Thus, semi-structured interviews convey a dialogic nature, allowing the interviewer and the interviewee to dialog, alternating moments of reciprocal exchange and moments of intimate, personal sharing from participants. Moreover, as interviews are scheduled and conducted, semi-structured interview guiding schema might be reviewed in light of participants’ feedback and preliminary analysis. In more details, the semi-structured interview guiding schema was developed from existing literature insights as well as by the team experience (CDV and IT) with the topic (see Supplementary Table S1). However, the design and development of the protocol was further refined through three pilot semi-structured interviews (not included in the analysis) that were realized with experienced veterinarians, already engaged in the topic, in order to assess the guiding questions and to inform the research team with strategic information regarding its accessibility and adequacy (69). Finally, all the semi-structured interviews in the study were conducted via the Zoom platform and lasted approximately 1H each. The first author realized all the semi-structured interviews, already having experience in adopting qualitative methods, conducting interviews, and working with the veterinarians. In order to evaluate the sample size sufficiency, a preassessment of data adequacy in terms of composition and size was performed to critically consider how saturation parameters found in prior methodological studies and sample size community norms could best guide and inform choices (70). However, instead of aiming for statistical generalizability, qualitative research focuses on depth by selecting information-rich cases that provide valuable insights into the phenomenon being studied (61). Finally, consistently with the analytic method (see below), the collection and analysis are carried out concurrently, requiring a certain degree of uncertainty (71). In the case of the present analysis, a saturation benchmark started to emerge after the 16th interview and the power of information gradually decreased after that. Coherently with the analytical framework, codes and preliminary themes were recurring after 16 interviews and we decided to run four more interviews to monitor saturation and determine if newer, unexpected and unrelated insights emerged. All 20 interviews were considered for analytical purposes. Our sampling strategy aligns closely with the concept of information power, which evaluates sample adequacy based on the richness of the data provided relative to the study’s objectives, rather than fixed numerical rules. Information power is determined by factors such as a narrow study aim, high sample specificity, strong theoretical grounding, and rich dialog quality. In this study, the specificity of the sample (veterinarians working with companion animals) and the depth achieved in the dialogic interviews (rich dialog) provided high information power. The emergence of saturation at 16 interviews, confirmed by the final four, indicated that sufficient information power had been achieved to adequately address the research questions.

The study was conducted in accordance with the Declaration of Helsinki and the code of conduct outlined by the American Psychological Association. For this research, institutional review board approval and ethical clearance were obtained by the Research Ethical Committee of the University of Padova (reference: 1093-b). All participants gave their written informed consent for inclusion before they took part in the study. The interviewer shared informed consent and privacy documentation through email. Data were collected between November 2024 and March 2025. All interviews were audio-recorded and transcribed verbatim for analyzing purposes. To ensure linguistic and semantic accuracy and quote fidelity, researchers translated the findings from Italian to English with the guidance of a native English speaker.

### 2.3 Participants

Given the research design, its aim and questions, the study adopted a non-probability sampling method to gather participants, combining purposive and convenience strategies (72). Using purposive sampling, we defined the target profile (general practice veterinarians working with companion animals in Northeastern Italy with ≥1 year experience). Then, through convenience (snowball) sampling, we recruited participants via social media posts (Facebook, Instagram, and LinkedIn, covering for most diffused personal and professional social networks in Italy) and referrals (73). Potential participants could contact researchers through first author’s email, expressing their willingness to participate. A total of *N* = 20 participants were interviewed. A total of *n* = 8 male veterinarians and *n* = 12 female veterinarians composed the participants’ group. The mean age was 42 and years of experience ranged from 2 to 40 (see Table 1). To ensure privacy, fictitious names have been assigned and used in the report.

**Table 1:** Participants’ sociodemographic data on years, years of working experience, and gender.

Fictious name	Years	Years of experience	Gender
Barbara	29	5	F
Erika	37	10	F
Giorgio	52	33	M
Michela	34	9	F
Gabriele	64	37	M
Andrea	29	3	M
Elena	44	18	F
Samuele	52	18	M
Giovanna	33	8	F
Maria	39	11	F
Ludovica	31	7	F
Gabriella	31	3	F
Alice	47	21	F
Luca	63	33	M
Mariano	48	40	M
Raffaele	44	15	M
Michele	30	5	M
Olivia	47	19	F
Rossana	36	9	F
Alessandra	58	30	F

### 2.4 Data analysis

This research implemented a Reflexive Thematic Analysis (RTA) approach to the qualitative analysis of textual data (74–76). The RTA coherently fits with the epistemological background informing the study’s methodology. RTA considers the researcher an active agent involved throughout all the research phases, drawing on pre-existing knowledge, assumptions, sensibilities, and perspectives that might enrich the analysis; at the same time, RTA values participants’ experiences as narratives emerging from a dialog intersecting multiple dimension. Addressing and engaging researcher involvement is cornerstone in RTA, as it helps the researcher gain awareness on their perspectives and provides complex connections and insights, guiding the depth of the analysis (75). Moreover, RTA theoretical and methodological standpoint is consistent with contemporary epistemologies issuing the centrality of textual data of indicating both personal accounts and broader social dynamics (76). The strength of RTA lies in considering data as being both subjective (e.g., personal) and intersubjective (e.g., interpersonal) (see also below).

Specifically, the analysis employed a recursive, iterative, abductive analytical approach, combining deductive coding (codes being selected and defined before the analysis, derived from pre-existing literature, knowledge and theories; i.e., top-down) and inductive coding (codes being identified, defined, and refined as the analysis proceeds; derived from participants’ vivid words and insights; i.e., bottom-up). In qualitative research, abduction means beginning with some theoretical concepts (deductive), but remaining flexible to develop, modify, or create new codes as unexpected patterns and insights arise from the data (inductive). This approach is iterative and cyclical—researchers continually move back and forth between theory and data, refining codes and themes to produce robust, contextually grounded explanations (77). The analysis adopted computer-assisted qualitative data analysis software (CAQDAS) (78), specifically Atlas.ti (79) version 25 to host interviews transcripts, allow codes generation, storage, and organization, support the researcher in grouping codes and quickly retrieve participants’ quotes. Thus, all the audio-recorded interviews were transcribed verbatim and input into Atlas.ti. The data collection and analysis were realized by CDV, who performed all the interviews and, thus, started the process of analysis. Thus, accordingly the analysis began from the first interview. The first phase of familiarization requires reading several times all the interviews, taking notes and starting to look for patterns. During the first phase, though, CDV and IT could dialog and confront themselves on what interviews were informing or indicating. In that sense, the second author could provide a more distant perspective. The second phase of coding instructs the researcher to code all relevant portions of the text, generating labels. The coding phase ends with the researcher reviewing all deductive and inductive codes, looking for recurring patterns, discrepancies, similarities, merging together similar codes, removing irrelevant ones, or modifying codes. After the second phase, CDV presented a preliminary codebook to IT and they discussed it and assessed coding quality by reviewing all quotations. Once codes were grouped in categories or families of codes, functioning as intermediate, guiding layers between coding and themes, the research group met to build themes that hermeneutically help to understand the set of data.

#### 2.4.1 Reflexivity and positionality

This research design stems from our commitment to situated, context-sensitive psychological inquiry, rooted in critical social psychology. We engage in interdisciplinary efforts to expand the literature’s observational and hermeneutic capabilities, particularly concerning underrepresented professional and emotional meaning-making. We ground this study in the conviction that professional testimony is not mere data but an epistemological act of agency. We view psychosocial research as a broader social endeavor that creates ongoing spaces for reflection and experimentation. Therefore, our aim was always transdisciplinary, asserting that understanding professional stress requires challenging traditional disciplinary boundaries beyond psychology. We maintained a reflexive stance throughout our analysis, aware that our own disciplinary positions, values, and experiences shaped our interpretations. Both authors are social and clinical psychologists and psychotherapists who approach mental health via a social and community-based model. We collaborate closely with clinical and academic veterinarians to share strategic knowledge and inform practice. During data collection, we invited participants to contextualize their perspectives relationally, deepening their narratives to include other agents, and we concentrated on the subjective experience emerging from intersections. Consequently, our data analysis was inspired by conceptualizing personal experience in terms of interconnected layers and dimensions, never losing the dialectic between the individual and the social (Table 2).

**Table 2:** A summary of the themes, definition and example of quotes.

Themes	Definition	Example of quotes
Fragmented Community of Practice	Narratives referring to experiences of competition, conflict with colleagues or feelings of lacking professional unity within the category	“*The real problem is also the relationship with colleagues […]. Everybody feels better than anybody, and thus there is no cooperation*”
Socioemotional Delegitimization	Multi-leveled experiences and micro-episodes of devaluation in own authority, knowledge, and feelings from society, clients, and social network	“*Even more clients treat me like I am a mere problem-solver*”
The Hidden Weight of Changing Clients in Veterinary Care	Experiential overlapping between clients’ new demands and veterinarians’ functional, invisible response	“*I have become both a general medicine practitioner and a psychologist, as I listen to clients’ complaints over their health*”
From Vocation to Unexpected Versatility: Imagination and Adaptation	Subjective work modification and adaptation of professional imaginary following practical experiences	“*Perhaps I was too naïve, or at least that is what some colleagues say to me. My expectations were completely different; I did not expect clients to have so much space besides compliance*”
Emerging Sensibilities, Expanding Responsibilities: A Moral Field	Experiences of personal and professional pressures arising from new ways of relating to the veterinary medicine, care and non-human animals	“*Back at my time, you just lived with your pet in your home; they would not be allowed inside. You had a different relationship; paradoxically, more domestic*”

## 3 Results

Through the Reflexive Thematic Analysis (RTA), we generated five themes: “Fragmented Community of Practice,” “Socioemotional delegitimization,” “The Hidden Weight of Changing Clients in Veterinary Care,” “From Vocation to Unexpected Versatility: Imagination and Adaptation,” “Emerging Sensibilities, Expanding Responsibilities: A Moral Field.”

The themes developed in the study span across several dimensions of veterinary experiences, capturing the complex and dynamic aspects that characterize the clinical work with clients and patients. The analysis reveals an interconnected landscape of structural, environmental, contextual, relational, and personal factors. Within this framework, elements such as the relationships with colleagues, perceived social and external neglection, interactions with clients, the personal representation of the professional identity and ethical dilemmas emerge as key psychosocial challenges. Taken together, these witnesses construct the web of profound threats to the mental health and overall well-being of veterinary professionals.

### 3.1 Theme 1: fragmented community of practice

Across all career stages, participants described a professional community marked by competition and a lack of mutual support, where the safeguarding of individual economic, career interests often act as a primary driving force. All the instances pointed to a shifting economic, work, and organizational model of veterinary care—one increasingly shaped by private corporations, medium-to-large clinics, and a market-oriented logic of personal business or enterprises. This has resulted in several episodes of miscommunications, conflicts, and litigations between colleagues, all of which contribute to the erosion of a coherent and collaborative professional identity. The words from Barbara (29 years, 5 years of experience) introduce the topic with much emphasis:

“It is a race to win over clients. You need to show yourself, advertise and promote your ‘brand’—sometimes even exaggerating the true reality of your capabilities. I know many colleagues who speak badly of their fellows just to protect and maintain their network of clients. And there are not limits to that; friends I studied with are struggling to stand out among so many of us. Every medium seems to be fair game. It’s hard for someone like me—I’m humble, insecure, and I don’t like showing off or overstating my abilities.”

Barbara’s words capture a broader dynamic that is governing health professions and its market, with the result that many professionals find it hard to position themselves—either due to a lack of business training or personal discomfort with self-promotion. Even though she is within a more mature professional phase, Erika (37 years, 10 years of work) similarly described a community which is increasingly moved by satisfying the client rather than caring for the patient.

“There is a lot of pressure coming in this shattered, fragmented community of practice, where controlling institutions fail to ensure adherence, the respect of clear, common guidelines, and I would say a common work-ethic. I know that if I refuse to satisfy a client’s demand because I think that it is not pertinent for the companion animal’s health, they will reach out for other colleagues. And they will surely find one adapting to their needs. Because, in the end, it is the owner that pays, and you need to bear that in mind.”

Also, Giorgio (52 years, 23 years of experience) noted a shift toward a fragmentation within the community, resulting in several, interconnected consequences. Financial strain is reshaping collegial relations and eroding long-standing professional norms:

“Among the several factors, the economic exposure of veterinarians is resulting in not transmitting, to other colleagues, difficult cases or situations which exceed one’s own competences, but rather in keeping it. Differently from a while ago, the economic profit and margin of the profession is becoming thinner and thinner. This is bad for the veterinarians themselves, because on the long run it can impair your sense of identity and the beauty of your work, forcing to submit everything under materialistic values; it is also negative, however, for the client, which puts their faith in us, and finally for the whole category, which prefers to take care of private and own business over communalities, networks, and exchanges.”

Together, these accounts illustrate how economic pressures and professional individualism are contributing to a fragmented, competitive field—where collaboration gives way to self-preservation and where ethical tensions become daily realities.

### 3.2 Theme 2: socioemotional delegitimization

This theme gathers participants’ emotionally charged experiences of delegitimization of their work in the cultural (ie, the perception of their work as it is formed through broad representations), environmental (ie, from their clients), and personal domains (ie, from their network of people). Veterinarians report feeling undervalued in their technical expertise, misunderstood in their emotional investment, and misrepresented in the public imagination. Delegitimization emerges both as a pervasive cultural atmosphere—a kind of invisible structure shaping how veterinary work is perceived—and as specific, emotionally fraught interactions in which their professional status is questioned or dismissed. Michela (34 years, 9 years of experience) on that note: “*I would like to be considered a doctor. I am not the villain who asks you for money to treat an animal. To me, she/he is a patient.*” Or Gabriele (64 years, 37 years of experience) succinctly said: “*It’s important to return to viewing animals from a more scientific perspective—to help people understand that it is not just about cuddles and cute outfits.*” Participants described how their role is often viewed through a sentimental or transactional lens, where compassion is expected to override compensation, and where medical authority is reduced to mechanistic task execution. This is particularly evident in client interactions, where veterinarians are often seen not as healthcare providers, but as passionate animal lovers whose services should be discounted or emotionally flexible. Andrea (29 years, 3 years of experience) shares an interesting interconnection between the previous and following theme:

“In my experience, the figure of the veterinarian is often not respected by clients. Many owners adopt the attitude of demanding, almost arrogant employers, showing little regard for our professional boundaries. They expect us to set aside our personal lives for the well-being of their animals—as if we didn’t already care deeply about them. At the same time, there’s too much competition among colleagues. Instead of collaborating for the good of the animals we care for, everyone tends to stay confined to their own area. For example, if a neurologist notices an internal medicine issue during a consultation, they often remain silent because ‘it’s not their responsibility.’ This lack of communication and teamwork ultimately harms both the patient and the profession.”

Moreover, Elena (44 years, 18 years of experience), during the interview, in multiple moments, highlighted how affective manipulation and economic pressures collide in her clinical work:

“I love animals, but this is still my job, and you need to pay me for what my work is worth and most importantly for what it cost in the past during my training and still costs me. They, the clients, think that because you are the ‘doctor of the animals’ and you chose this job for passion, you can amend and make discount every time, like my medical performance is easier or less technical than that of human doctors. I am a medical doctor, not a social assistant nor a volunteer.”

Her experience points to a double standard applied to caregiving professions: passion becomes a reason to diminish professional legitimacy and to undervalue technical skills, resulting in deep discomfort, especially around moments of payment. Elena’s experience highlights social delegitimization in terms of the economic and financial aspects of her profession (as she later shares that she has come to hate the moment of payment), as well as micro-forms of affective manipulation, that can become intrusive and destabilizing. In a similar vein, Samuele (52 years, 18 years of experience) describes all the struggles and efforts that have characterized much of his professional career, recounting all the time dedicated to legitimizing his role.

“There is this common-sense belief that veterinarians should work only for the sake of their patients’ health and well-being, leaving aside any other form of gratification. Moreover, there is a very naïve view on most of companion animals’ pathologies, and your job is valued as simple and mechanistic. But this is not mathematic, and clients need to acknowledge that. Any trouble, complications is not tolerated. Clients do not usually understand that animals’ anatomy and physiology is incredibly complex, more complex than they can imagine. My work is highly technical and requires years and years of experience and expertise.”

Lastly, Giovanna (33 years, 8 years of experience) shares neglecting experiences and instances of delegitimization coming from her personal and relational network:

“You know, as there is the so-called disenfranchised grief for those who lose a companion animal, similarly I feel that most of my friends fail to acknowledge that sufferance and grief over patients’ and clients’ sorrow can be extremely hard to deal with also in veterinary medicine. That you can come home with a lump in the throat because you witnessed the last moments of a beautiful bond between a human guardian and the companion animal; or because you cared for a beautiful living creature until their last moments of life.”

Giovanna’s words expand the theme’s circle toward a form of experienced psychological invisibility—a moral and emotional burden that remains unacknowledged both within and outside the profession.

### 3.3 Theme 3: the hidden weight of changing clients in veterinary care

The relationship with clients was a major, recursive topic among all the interviews. The theme is multifaceted, and its foundations branch out like the roots of a tree. However, the designated denomination “The Hidden Weight of Changing Clients in Veterinary Care” aims to capture two interconnected feelings: firstly, the emotional work contemporary veterinarians need to perform in order to welcome, listen, and understand their clients; secondly, the progressive novelties clients bring to the relationship, everyday breaking representations of what means to be a client, creating an unstable ground for veterinarians. Thus, the theme aims to highlight the emotional labor involved in navigating relationships with clients; the changing nature of the expected client, shaped by misinformation, unrealistic expectations, and emotional projection; and the evolving burden veterinarians carry in absorbing not just medical complexity, but human distress and inadequacy. In veterinary medicine, veterinarians are in constant direct contact with both clients and patients, without any mediating structures—no social buffer exists. This absence of modulation exposes veterinarians to continuous scrutiny. Owners frequently seek explanations, justifications, and sometimes even engage in conflict. This direct exposure is a major source of stress as makes the work constant under observation and open to judgment from individuals. Maria (39 years, 11 years of experience) shares:

“The relationship with clients is one of the most demanding aspects of my profession. They come expecting solutions to complex problems during a simple clinical consultation, without offering any additional information or effort. When I draw upon the vast body of medical knowledge, they ask me to simplify it. They want and expect everything—answers to every question, even predictions about the future. Some people neither understand nor want to understand. Many take on dogs they cannot manage and handle them poorly—and we end up putting ourselves at risk. People become aggressive when you say no and set boundaries.”

Giorgio (52 years, 23 years of experience) expresses frustration over the widening gap between the availability of information and the quality of knowledge among clients. Rather than fostering awareness, this information overload has produced indifference, resistance to listening, and an alarming dismissiveness toward professional guidance.

“The increase in available information has not led to greater awareness or education. Animal care today is often handled with striking ignorance. There is an overwhelming flow of fast, superficial information, yet people seem to listen less and less. The most common reaction is indifference—‘You cannot save them all.’ And it is disheartening, because the animal is not to blame”

Always Maria (39 years, 11 years of experience) reclaims the functional differentiation that is investing her role alongside an invisible work that goes beyond the care of the animal toward the creation of a relational environment where the client can feel understood: “*They kind of confess to you. And I listen, also sometimes I try to give some tips, I try to help. Perhaps, it is useful for the animal’s health to have an owner which is more “centered.” But I do now know, it does not feel like my job.*”

Ludovica (31 years, almost 7 years of experience) highlights the emotional intensity of client–veterinarian relationships, particularly the experience of becoming a container for clients’ distress and anxiety:

“They tell you everything—bring medical reports, share intimate personal stories. It is as if they unload all their anxiety onto me. I try to contain it—sometimes I manage, sometimes not. Some of that stress follows me home, where I find ways to let it out. There are heavy days, but over the years you learn to manage these situations and release the emotional burden. You also learn to assign less weight to certain dynamics. When someone tries to blame you for something that’s not your responsibility, I have learned how to defend myself. It is a particular kind of stress. It was not like this in the beginning—especially when I was working for others. You always lived in fear that saying one wrong word might cost you your job.”

Veterinarians in this study were frequently expected to absorb and manage emotions that go far beyond the clinical encounter. This emotional spillover is not only difficult to regulate, but it also follows practitioners into their personal lives, blurring boundaries and contributing to cumulative stress.

### 3.4 Theme 4: from vocation to unexpected versatility: imagination and adaptation

The following theme stems from participants’ narrating a dense multitude of episodes where they shared a set of actions, tasks, performances related to their work that they did not expect to do, nor did they think were part of veterinary care. In other words, the theme aims to highlight the experience of facing a misalignment between professional training/identity and the practical realities of veterinarians’ work, leading to role expansion, identity strain, and ongoing adaptation. The continuity with the precedent theme is interesting, though this theme captures more the emotional and cognitive destabilization that follows the awareness that the idealized picture of the profession will have to change, like in these quotes: “*I find myself working with the bipedal companion.*” This caustic statement captures a role inversion: rather than engaging solely in the animal’s medical needs, the veterinarian becomes entangled in the human guardian’s psychological and affective landscape. Gabriella (31 years, 3 years of experience) did not expect that, and she finds it profoundly difficult to come to terms with the fact that she has to rethink her profession from the inside:

“I truly did not expect that. It is like I have to review the whole image I have of being a veterinarian. I thought I was going to work for the animal welfare, in close proximity and contact; most of the time, however, I find myself working for the compliance of the owner, doing a difficult job. It is a little bit harder than I thought.”

Samuele (52 years, 18 years of experience) likewise finds himself redirecting attention to the animal’s needs, even when this entails a difficult conversation with the client.

“This animal is unwell, suffering, and there is no further treatment we can offer. You can see it—I can see it. But sometimes the owner says, ‘But it hurts me.’ And I answer, ‘I am not concerned with how this affects you emotionally. Your pain is your own—it is not the animal’s. You need to focus on the animal, not on yourself.’ I say it plainly: ‘If you do not believe me, go home and watch your animal as it suffers. Understand that it is not the animal who is causing your pain—it is your fear of your own suffering. And that fear is preventing you from truly seeing the situation.’ In the end, I am speaking to you, but the pain is not on the table—the animal is.”

### 3.5 Theme 5: emerging sensibilities, expanding responsibilities: a moral field

This theme brings together participants’ accounts on ethical challenges and experiences of moral distress in everyday clinical practice. Its distinctive focus lies not in isolating specific sources of distress—such as end-of-life decisions, treatment options, or client decision-making—but in exploring what makes the work burdensome from a holistic experiential standpoint. The theme seeks to uncover a common, shared ground across individual and unique accounts of distress.

Participants consistently highlighted the expanding range of situations in which a moral stance is expected—or even required. Many, particularly those with longer careers or senior roles, emphasized that they have always embraced an intense sense of responsibility and upheld high ethical standards throughout their practice. What has changed, however, is the emergence of new knowledge (from medical-technical side as well as from clients’ one) and heightened sensitivities concerning human–nonhuman animal relationships, both ecologically and locally. These evolving perspectives, involving human guardians and their companion animals, now tend to permeate and intersect every aspect of clinical decision-making. Thus, regardless of personal judgments over new relational patterns between humans and non-human animals, new affective atmospheres surround the clinical environment.

From participants’ words, the common ground regards the multiplication of areas where a moral position is expected, if not necessary. All participants, especially those with more years of work or occupying seniority positions, recounted to have always been truly responsible as well as to have inspired their practice to high ethical standards and moral values from the beginning. What has changed, however, is that new knowledge and sensibilities invest the human-nonhuman animal relationships, ecologically, and locally, human guardians and companion animals, making ethical and moral reflection fundamental to every step of clinical practice. With the rise of new sensibilities come new forms of professional responsibility—fragmented yet interconnected moments that collectively reveal the evolving sociocultural landscape of and around veterinary care. In Alice’s words (47 years, 21 years of experience):

“I think there is another crucial factor to consider: the way we relate to animals has changed. Owners now expect something different—they tend to see their animals as full-fledged members of the family. But it is not just the owners—veterinarians are part of this shift too. The sense of responsibility toward an animal today is very different from what it used to be. When I care for a puppy—or any animal, really, regardless of age—it is as if I am caring for a child. That is how the owners refer to them, and you end up doing the same: ‘How is your little one doing? How is your girl?’”

She continues: “*However, you also begin to perceive the animal as a member of the human family. And so, if before the dog was the dog, now the dog is no longer the dog. Yes, you treat it as the dog.*” From these words, the phenomenological landscape permeating the professional practice, the clinic, the ambulatory seems very permeable to what happens in the outside social contexts. Luca’s (63 years, 33 years of experience) words capture the feeling of pressure coming from new sensibilities around veterinary care, which also help explain some work-related trends about diagnostics:

“And that is why I think on the one hand diagnostics have exploded, so everything that can help you to get to the diagnosis, to map every moment, to keep track of everything. But the bottom line is you, the outcome of a blood test, because you are the one interpreting it, it is not the outcome of the ultrasound, because you are the one putting the pieces together. It is almost like wanting to say ‘Okay, I have that test that tells me this, so it is the test that says it’ and I say it to remove a little bit of pressure from another responsibility.”

Mariano (68 years and 40 years of experience) adds an important piece to the puzzle of understanding how professional work and society are strictly interconnected in the understanding of a wide array of experiences in everyday practice:

“In my opinion, veterinarians need to protect themselves from those clients who arrive, place the animal on the table, and then step back as if saying, ‘Change the spark plugs,’ as if they were at a mechanic. It is essential to help them understand that the animal is their responsibility at all times—even when it is under veterinary care. They must participate actively; treatment is a collaborative effort. The veterinarian does not simply solve the problem; the solution must be reached together. This is a message we constantly try to convey, although it is increasingly difficult because people’s mindset has changed. In the past, animals were an integral part of the household, whether inside or outside the home, there was continuous contact. People raised animals with an awareness of their existence and needs, and they understood that care was necessary in order to achieve results. Today, many people have never had animals, and suddenly they acquire a puppy as if it were a PlayStation or a new car—an attractive object to be shown off.”

## 4 Discussion

The present qualitative study identifies five interconnected themes that illuminate the complex psychosocial landscape of veterinary practice in Northeastern Italy: Fragmented Community of Practice, Socioemotional Delegitimization, The Hidden Weight of Changing Clients, From Vocation to Unexpected Versatility, and Emerging Sensibilities. These findings confirm and extend a growing body of international research on mental health challenges—reporting elevated rates of depression, anxiety, burnout, and suicide risk (7–10)—through the analysis of profound, emotional experiences and narratives.

However, by adopting a psychosocial stance, this study shifts the focus from individual-level vulnerability to the broader lived experience of veterinarians, revealing how psychological malaise is shaped by deep structural, cultural, and interpersonal transformations in the profession, and thus enriching the field with an ecological perspective. At the heart of this analysis lies the recognition that veterinary practice is undergoing a profound metamorphosis, driven by shifting societal expectations, changing human–animal relationships, cultural models, and evolving workplace structures. In other words, the sociocultural image and mandate assigned to veterinarians, and traditionally biomedical occupational groups in general, is changing, bringing along a vast field of psychological tensions and strains that point to a health-related urgency as well as to a vital, capable and resilient profession.

Findings of the study provide crucial qualitative depth to quantitative findings consistently identifying client interactions as a primary source of professional stress. While it echoes similar findings pointing at client relationships as having a fundamental role in shaping negative experiences (80, 81), a profound emotional labor veterinarians perform in client relationships clearly emerged (82–84). However, previous research did not capture the systemic nature of these challenges or their connection to broader sociocultural transformations in human-animal relationships. Our participants suggest that client-related stress is not merely an interpersonal issue but reflects fundamental changes in the social contract between veterinarians and society. On a similar line, while literature has acknowledged professional identity challenges to mental health outcomes (4, 6, 85) our findings reveal how market-oriented logic is fragmenting the veterinary community. Systemic issues—economic pressures, professional fragmentation, and societal delegitimization—may be equally or more significant in understanding veterinary distress as other more traditional individual-based variables, calling for a stronger implementation of mesosystemic analysis (86). It is not just a matter of intra-professional stressors (87), but these results indicate a need to examine how broader cultural and social patterns influence the very nature and function of the veterinary profession. Indeed, in many Western contexts, companion animals are now regarded as full-fledged family members, and the emotional intensity of these bonds increasingly mirrors that of human-to-human attachments. This cultural shift has expanded the veterinarian’s role beyond clinical care to encompass emotional labor, ethical mediation, and even grief support (53–56). As highlighted in this study, veterinarians are expected to manage not only animal patients but also the psychological needs of human guardians, often navigating conflicting demands and intense affective consequences. These findings resonate with emerging conceptual frameworks such as affective ecology (88) and posthumanist critiques of biomedical reductionism (89), which call for a reframing of care professions as embedded in multispecies relational fields, thanks also to feminists’ thoughts (90). Within this new ecology of care, the veterinary encounter unfolds as a triadic configuration—veterinarian, animal, client—each carrying symbolic and emotional weight. Such complexity places heavy demands on practitioners, who often lack the psychological and communicative training to navigate these terrains effectively. Participants in this study describe a growing tension between “humanity and technicity,” reflecting the difficulty of reconciling ethical vocation with clinical standardization and market pressures. Indeed, a notable finding emerging from this research is the increasing neoliberalization of veterinary medicine. As the profession shifts from individual practice to corporate and franchised service models, veterinarians report a loss of autonomy, a rise in competitiveness, and the marginalization of collaborative values. Business skills, visibility, and brand management are often prioritized over clinical expertise and ethical reflection, intensifying feelings of inadequacy, isolation, and misrecognition. These pressures also foster a fragmented professional identity, where emotional labor is underacknowledged and socioemotional challenges are internalized as personal failures rather than systemic stressors. Centrality of identity, moreover, was also noted in one qualitative study carried in South-African context (91). Taken together, these insights call for a critical rethinking of how veterinary training and organizational structures address mental health. As additional scholars have argued from different perspectives (92–94), it is time for veterinary medicine to mirror its human counterpart by integrating humanity-informed, psychosocial approaches into education and care. Strengthening veterinarians’ relational, communicative, and reflective capacities through targeted curricula and support systems is not only a matter of well-being but also a strategic imperative for sustaining the ethical and emotional integrity of the profession.

We must acknowledge certain boundaries of this research. The study is confined to a specific sample: 20 veterinarians working exclusively with companion animals in Northeastern Italy. This geographic and professional specificity inherently shapes the findings. Consequently, while statistical generalizability is not the goal of this qualitative work, this context-bound nature must be considered when assessing the transferability of these insights to other cultural contexts, healthcare systems, or different veterinary practice types within this very stratified profession.

Furthermore, the employment of convenience and snowball sampling through social media may have resulted in a sample that does not fully capture the diversity and plurality of veterinary experiences. This is a particularly relevant consideration given that representing this breadth of meaning-making is a core aim of our critical perspective.

## 5 Conclusion

Future theoretical and empirical perspectives should consider that veterinary medicine is undergoing a significant transformation, gradually expanding beyond its traditional clinical/technical focus to encompass broader public health and socio-caring functions. This shift is driven by the evolving expectations and emotional investment of clients, who increasingly perceive their animals as family members. At the same time, cultural contradictory tropes of anthropomorphism and antispeciesism combine together into a cultural assemblage structuring in plural ways human and non-human animal relationships. As a result, the veterinary profession is now called upon to respond not only to animal welfare from a physical health concern, but also to psychological and sociocultural aspects of the human–animal relationship. This new dynamic reflects a profound cultural change in how society relates to non-human animals, attributing to them emotional significance and moral consideration. Consequently, veterinarians are often confronted with situations of emotional distress, grief, or ethical tension that lie beyond the scope of standard medical expertise, demanding new relational and communicative skills. At the same time, broader shifts in healthcare systems are altering service delivery models and redefining professional roles. These developments call for a rethinking of veterinary medicine’s contribution within a wider global health framework, encouraging an integrated, interdisciplinary approach that acknowledges the interconnectedness of human, animal, and environmental well-being. In this context, an ecological model of care invites healthcare professionals to reconsider the individualistic logic that has traditionally guided clinical practice, shifting the emphasis away from the technical monopoly of care and toward the construction of systemic functions, in which both professional and non-professional actors play a role. By focusing on the totality and integrity of the illness and care experience of the patient or client, this model challenges the still-dominant linear and vertical delivery of services—represented by the “A to B” logic of healthcare provision—and instead offers a community- and network-based resource for health systems. It promotes the integration of services and professional expertise across a given territory, establishing a meta-model that enables continuous knowledge exchange and acts as a protective factor against the primary occupational risks faced by veterinary professionals.

This study extends current knowledge by revealing how broader cultural shifts (e.g., pet owners’ expectations, corporatization of clinics) are fundamentally altering veterinarians’ roles and well-being. These qualitative insights provide context to known statistical trends, demonstrating that interventions must target not just individuals but also professional communities and societal attitudes.

### 5.1 Implications for practice

The qualitative insights from this study can inform important practical implications at several levels.

In terms of veterinary education programs, curricula should include training in communication skills, positive psychology and emotional resilience, and interpersonal competences. More than that, as experimented in other fields, training should also have an experiential base, facilitating a translational, applicative value of theoretical insights. Given the evolving client–veterinarians relationships and heightened emotional labor, veterinary students and practitioners need a technical preparation for managing interactions with client and orient consultations. Integrating modules on client communication, conflict resolution or de-escalation, and self-care into training can better equip veterinarians to handle interpersonal challenges. At the level of professional organizations, stakeholders and policy development, policies should reflect an ecological, multi-level approach. A multi-faceted approach is essential. At the institutional level, organizations should provide vital mental health resources for veterinarians, such as confidential counseling services or group-based supervision. Simultaneously, they must formulate clear guidelines that actively promote a healthy work-life balance and outline protocols for responsible client interactions. On a broader scale, policymakers should develop public awareness campaigns to counteract the common misunderstanding or delegitimization of the profession’s socioemotional challenges. Finally, individual veterinarians can apply these insights in their own clinical practice by consciously fostering more cooperative relationships with both clients and colleagues. The study’s participants stressed the importance of involving clients in treatment decisions as well as setting boundaries to ensure shared responsibility for animal care. Practitioners might implement regular debriefings or peer support groups within clinics to discuss difficult cases, thereby reducing individual burden and moral stress.

## Data Availability

The datasets presented in this article are not readily available because of privacy restrictions. Requests to access the datasets should be directed to ciro.devincenzo@unipd.it upon reasonable request.
