# Supplementary material for: Ecologies of care: qualitative insights from a psychosocial study on veterinarians’ experiences in Northeastern Italy
Source: Front Vet Sci. 2025 Dec 4;12:1650809. doi: 10.3389/fvets.2025.1650809 (PMC12713320; doi:10.3389/fvets.2025.1650809)
Supplement: Supplementary file 1 [file Table_1.docx]

Supplementary Material

# Appendix A – Semi-structured Interviews Guiding Schema

| Interview moment | Illustrative Questions | Aim/s |
| --- | --- | --- |
| Beginning: Introductory grounding | - How old are you and since when have you worked as a veterinary? - Where did you study? Did you also pursue a postgraduate specialization or course? | - To introduce ourselves to each other, share and explain research aims - To break the ice and establish a comfortable, trustworthy situation |
| Background: Context information | - In a typical day, how many clients and patients do you meet? - How was today/yesterday working day? Could you describe it? - Who are the people you interacted with the most at work over the past three days, and what happened in those interactions? | - To approach everyday practice closer and orient participants’ narratives toward their experiences - To map and identify core narrative elements anchoring veterinarians’ experiences |
| Figure: Emotions and experiences | - What are the most challenging moments in your daily practice, and how do they affect you emotionally? - How would you describe your emotional experience in your relationships with clients? - Which emotions do you find most difficult to process in your work, and in what ways do they influence your perception of your role or the profession? | - To explore the emotional landscape of veterinarians’ clinical practice, identifying situations that elicit psychological distress, ethical tension, or emotional saturation. - To understand how interpersonal dynamics influence veterinarians’ emotional well-being and their evolving professional identity. |
| Defusing and Reflections | - What do you see as the main challenges for your future work, and for the next generation of veterinarians? - If you were to describe your profession to first-year veterinary students, which aspects would you choose to emphasize? | - To explore participants’ anticipations and concerns about the future of veterinary practice, including systemic, emotional, and relational challenges. |
| Concluding remarks | - During this interview, did any particular feelings or emotions come up for you? - Is there anything else you would like to add that we haven't covered? | - To provide an open-ended opportunity for participants to share insights or experiences not prompted by prior questions, supporting a participant-led closure and enhancing narrative richness. |
